# The effect of co-administration of vitamin E and C supplements on plasma oxidative stress biomarkers and antioxidant capacity: a GRADE-assessed systematic review and meta-analysis of randomized controlled trials with meta-regression

**DOI:** 10.3389/fimmu.2025.1547888

**Published:** 2025-07-16

**Authors:** Mahdi Moabedi, Alireza Milajerdi

**Affiliations:** ^1^ Students’ Research Committee, Isfahan University of Medical Sciences, Isfahan, Iran; ^2^ Department of Community Nutrition, School of Nutrition and Food Science, Nutrition and Food Security Research Center, Isfahan University of Medical Sciences, Isfahan, Iran; ^3^ Research Center for Biochemistry and Nutrition in Metabolic Diseases, Institute for Basic Sciences, Kashan University of Medical Sciences, Kashan, Iran

**Keywords:** oxidative stress, meta - analysis, alpha tocoferol, randomized controlled trials, antioxidant status, ascorbic acid

## Abstract

**Background:**

There is no conclusion on the effect of vitamins E and C on plasma biomarkers of oxidative stress and antioxidant status. We conducted this meta-analysis to gain a clearer view of this area.

**Methods:**

We performed a systematic search in online databases using the relevant keyword combination. Randomized controlled trials that investigated the effect of vitamins E and C simultaneously, compared with control, on oxidative stress and antioxidant status biomarkers were included in the analyses.

**Results:**

A total of 17 trials were included in the meta-analysis, building a total sample size of 965. The dosage of vitamin E and C supplements varied from 54 to 536 and 250 to 1000 mg/d, respectively, across included studies. We found significant results for MDA [WMD: -0.38, 95% CI: -0.48, -0.28 µg/L, P <0.001], LP [WMD: -1.01, 95% CI: -1.49, -0.54 µg/L, P <0.001], TAC [WMD: 0.09, 95% CI: 0.05, 0.13 mmol/L, P <0.001], and GPx [WMD: 1251.74, 95% CI: 258.92, 2244.56 U/L, P = 0.013], but not for SOD [WMD: 16.69, 95% CI: -29.40, 62.78 U/L, P = 0.278]. Regarding subgroup analysis, only studies on unhealthy participants showed a significant effect on MDA [WMD: -1.62, 95% CI: -2.08, -1.15 µg/L, P <0.001] and TAC [WMD: 0.08, 95% CI: 0.03, 0.14 mmol/L, P <0.001], unlike LP, where significance was only observed in healthy adults [WMD: -0.41, 95% CI: -0.45, -0.37 µg/L, P <0.001]. Moreover, only studies in which a placebo was administered, supplementation of vitamins showed significant effects on MDA [WMD: -0.47, 95% CI: -0.58, -0.35 µg/L, P <0.001], LP [WMD: -1.28, 95% CI: -1.85, -0.72 µg/L, P <0.001], and TAC [WMD: 0.10, 95% CI: 0.05, 0.15 mmol/L, P <0.001].

**Conclusion:**

Our review and analyses revealed that a combination of vitamin C and E has a beneficial effect on oxidative stress biomarkers and antioxidant status.

**Systematic review registration:**

https://www.crd.york.ac.uk/PROSPERO/view/CRD42024590197, identifier CRD42024590197.

## 1 Introduction

Oxidative Stress (OS) is a state of an imbalance between oxidants and antioxidants in favor of the oxidants, which leads to a disruption of redox signaling and control and/or molecular damage (1). In this condition, the production of activated or reactive forms of oxygen (ROS) is much higher than the capacity of the detoxifiers available in the body for neutralizing them (2). There are multiple species of ROS including superoxide anion (O_2_^.-^), hydrogen peroxide (H_2_O_2_), singlet oxygen (1O_2_), hypochloric acid (HOCl), ozone (O_3_), lipid peroxyl (LOO.), hydroxyl radical (.OH), and reactive nitrogen species (RNS) like peroxynitrite (ONOO^-^) and nitric oxide (NO.); which all are responsible for OS occurrence (3). High generation of ROS leads to pro-inflammatory mediators and inflammation as a result. By this pathway, OS may cause cell death and tissue remodeling indirectly, potentially leading to further inflammatory diseases. It can also directly cause oxidation in lipids, proteins, or even nucleic acids, like gene mutation, which may lead to carcinogenesis (4).

Numerous factors are responsible for oxidative stress, such as high exposure to ultraviolet (UV) light, air pollution, ionizing radiation, certain chemicals and drugs, and lifestyle and nutritional factors (5). One of the most crucial dietary factors is the daily intake of antioxidants. Vitamin E and C as two of the most important antioxidants that act in fat- and water-soluble phases, respectively (6, 7). Ascorbic acid also restores α-tocopherol by reducing its residual which are called α-tocopherol quinone (8, 9). Both of these antioxidants neutralize oxidants by giving them their electron and converting them into less aggressive molecules (10, 11). However, there is no conclusion in this area, not all RCTs report the same result for the co-supplementation of vitamins E and C on oxidative stress and oxidant capacity, and the findings are inconstant. Some RCTs have shown a significant beneficial effect on plasma oxidative stress and antioxidant capacity biomarkers, while others have not. Interestingly, some RCTs have rejected the hypothesis that their being useful for the decline of those biomarkers. Surprisingly, no meta-analysis or systematic review was conducted on this crucial topic.

All in all, there is a need for a comprehensive meta-analysis combining all available data in this area and generating a conclusive result. Thus, we conducted the current systematic review and meta-analysis summarizing available evidence on the effect of vitamin E and C co-supplementation on plasma oxidative stress and antioxidant capacity biomarkers.

## 2 Methods

The current review study was conducted based on the PRISMA protocol for systematic review and meta-analysis reporting. The protocol is available on PROSPERO with the following registration code: CRD42024590197.

### 2.1 Search strategy

A systematic comprehensive search was performed in PubMed, Web of Science, and Scopus to September 2024 with looking for following keywords ((“Vitamin E” OR Tocopherol OR tocotrienol) AND (“Acid Ascorbic” OR “L-Ascorbic Acid” OR “Acid L-Ascorbic” OR “L Ascorbic Acid” OR “Vitamin C” OR Hybrin OR “Sodium Ascorbate” OR “Ascorbate Sodium” OR “Ascorbic Acid, Monosodium Salt” OR “Ferrous Ascorbate” OR “Ascorbate Ferrous” OR “Magnesium Ascorbate” OR “Ascorbate, Magnesium” OR “Magnesium di-L-Ascorbate” OR “Magnesium di L Ascorbate” OR “di-L-Ascorbate Magnesium” OR “Magnesium Ascorbicum”) AND (“Oxidative stress” OR malondialdehyde OR MDA OR Glutathione OR GSH OR “Total Antioxidant Capacity” OR TAC OR “total antioxidant status” OR TAS OR “nitric oxide” OR “antioxidant” OR “superoxide dismutase” OR SOD OR “glutathione reductase”) AND (RCT OR “Randomized controlled trial” OR “Randomized clinical trial” OR “Random allocation” OR “Random assignment” OR trial OR trials OR randomized OR randomised OR controlled OR blind OR blinded OR crossover)) in title and abstracts except for the concept determining study design which was looked for threw full-texts. To avoid potential missing (despite rigorous systematic search), we also screened the first twenty pages of Google Scholar using (tocopherol AND “ascorbic acid” AND oxidative) term combination and a simple search.

No limitation in any condition, such as time, language, study location, or journal, was considered during the process. To minimize the chance of missing any publications, we also reviewed similar articles in PubMed and the reference lists of relevant papers. All the references were imported to EndNote (version 21.3) for screening. Duplicate and unpublished studies and preprints were removed afterward.

### 2.2 Inclusion criteria

The following criteria were considered for inclusion of studies: 1- controlled trials with random allocation of participants in groups, 2- publications in which participants were adolescents or adults, 3- studies in which a specific tocopherol or tocotrienol and ascorbic acid were administered as a co-supplement, 4- papers that reported mean changes and standard deviations (SDs) of plasma oxidative stress and/or plasma oxidant capacity, or the required data to obtain them. If there were more than one publication on the same dataset, we only included the complete and highest-quality one. In case a study had two arms of intervention, each arm was considered separate and identified with the lowercase alphabet in the analyses. To avoid an increase in the power of the study with this condition, the number of participants in the control group was divided between each arm.

### 2.3 Exclusion criteria

We excluded observational-designed studies, including prospective cohorts, case-control studies, and cross-sectional studies. Moreover, we excluded review literature papers from any sets during the initial screening stages. Finally, we excluded trials without a control group and studies in which children were included as subjects.

### 2.4 Data extraction

Data extraction was performed by two separate and independent investigators. During data extraction, the following data was obtained from included RCTs: first author name, publication year, number and gender of subjects, mean age of participants, study design, baseline serum concentration and dietary ascorbic acid and α-tocopherol, the dosages of vitamin E and C, study duration, administration of placebo and its combination and characteristics, study compliance, confounding variables with were adjusted in the analyses, consumption of supplements or medication with an effect on oxidative stress or antioxidant status, study compliance, health condition of participants, and finally, oxidative stress and antioxidant status biomarkers with their mean changes and SDs or any possible data for obtaining them. If the data on the biomarkers were reported in different units, we converted them to the most frequent and common unit.

### 2.5 Risk of bias and quality assessment

All included trials were assessed using the Cochrane quality assessment tool for risk of bias version 2 (RoB2) (12). This tool contains seven domains: 1- random allocation, 2- allocation blindness, 3- selective reporting bias, 4- blindness of participants and personnel, 5- blindness of outcome assessments, 6- incomplete outcome data, 7- other sources of bias which in the current systematic review is “a low level of adherence to the study by participants”. If there was a methodological error in each domain of the study, the domain was given an H as “high risk”. If there was no defect for that domain, it was given an L as “low risk”. If there was insufficient information to determine, the domain received a U as “unclear risk”. To obtain an overall score, we gave values to L, H, and U as 1, -1, and 0, respectively. Each study could get a score between -7 and 7. A study with score of 3.5< was considered “Excellent” quality. If the score was ≤3.5, >0, the study was labeled “Good”. A score of more than -3.5 to 0 was named “Adequate”. Finally, if the score was -3.5 or less, the study was considered to have a “Poor” quality.

### 2.6 Statistical analysis

Mean changes of biomarkers with their SDs in the intervention and control groups were used to calculate the overall effect sizes. If a mean change was missing, we obtained it using pre- and post-intervention means and their SDs. When data was reported as the median and interquartile range (IQR), we converted the data to means and SDs using the method by Wan et al. (13). Standard errors (SEs) and 95% confidence intervals (CIs) were converted into SDs using the method by Hozo et al. (13). If α-tocopherol dosage was reported in the international unit (IU), it was converted into milligrams by multiplying the value in IU by 0.67. A random-effect model, which considers study variations, was applied to obtain overall effect sizes in overall and subgroup analyses. I^2^ statistics and Cochrane’s Q test were taken into account for the determination of heterogeneity between studies. An I^2^ > 50% and a P-value < 0.05 were considered significant between-study heterogeneity.

To find the potential sources of heterogeneity, subgroup analyses and meta-regression tests were conducted. Subgroup analyses were performed based on intervention duration (<8 vs. 8–24 vs. >24), study location (Asia vs. Europe vs. America), method of control (placebo vs. no placebo), health status (healthy vs. unhealthy), degree of study blindness (non-blinded vs. single-blinded vs. double-blinded vs. triple-blinded), and study quality based on RoB2 with given score (Poor vs. Adequate vs. Good vs. Excellent). Meta-regression was conducted on the effect according to the dosage of vitamin E and vitamin C (mg/day), the score obtained from RoB2, and the duration of the intervention (weeks). Sensitivity analyses were performed for each biomarker separately to detect the dependency of the overall results on each particular study. The potential publication bias was assessed using the Begg test. Stata 17.0 (StataCorp) was utilized for the analyses to be carried out. The significance level was considered based on a P-value < 0.05.

## 3 Results

The primary outcome of our systematic search consisted of 9697 records. After excluding 2670 duplicate references, 7027 records were left for initial screening. 6765 completely irrelevant papers were excluded based on title and abstract during the process, resulting in 262 studies for further evaluation. Between these records, 226 more records were excluded after a deeper look at the abstract and keywords. During the full-text assessment of the remaining 36 records, 3 animal studies (14–16) 5 papers with incomplete data (17–21), 7 references without acceptable control groups (21–27), and a study for the co-administration of other supplements was omitted from the final inclusion (28). Moreover, there was a duplicate study with the same dataset but in different journals and publication dates, and the older one was excluded.

**Figure 1:**
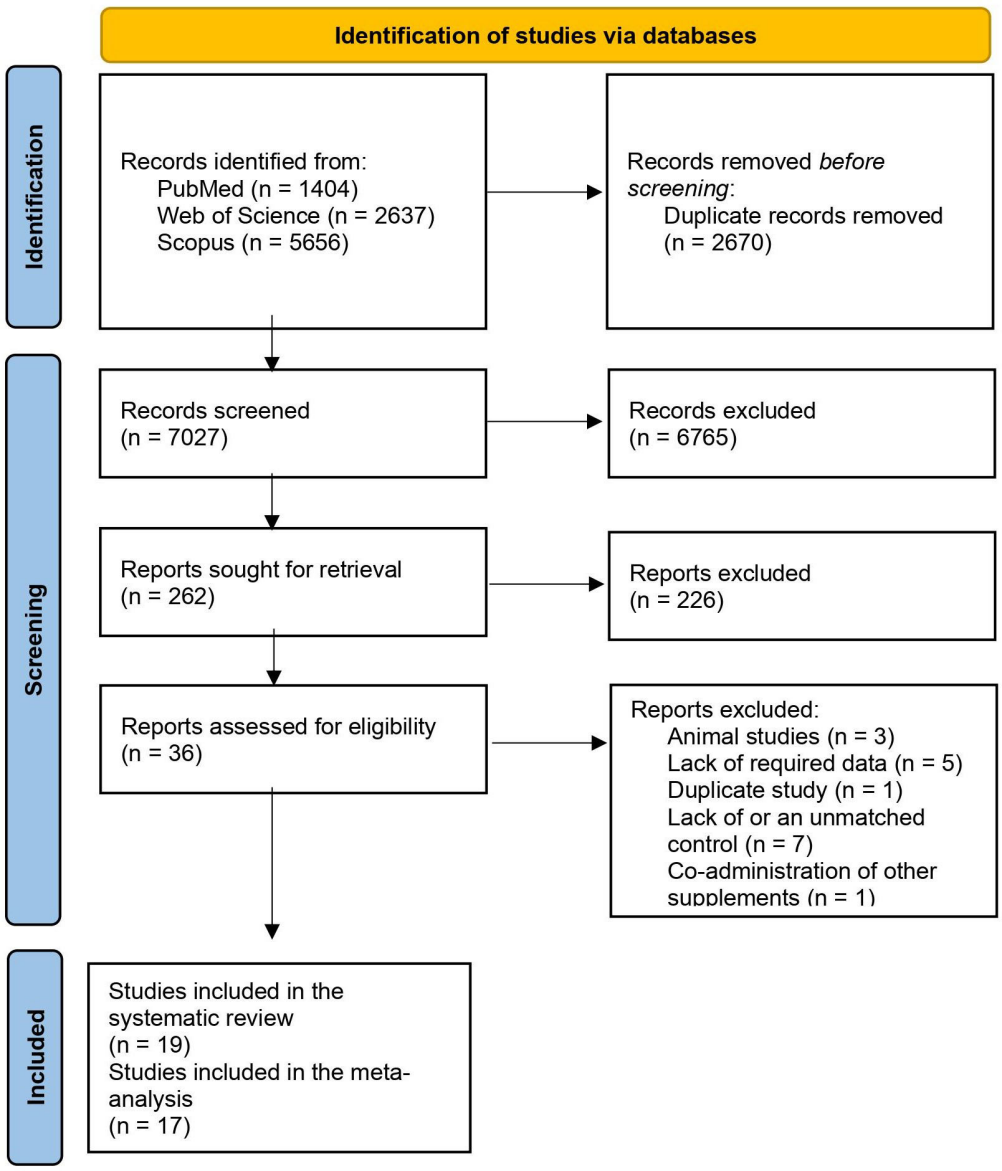
Flow diagram of the study selection.

After the screening and these exclusions, 19 trials met the inclusion criteria of the systematic review (29–47) and 17 RCTs were eligible for meta-analysis (29–37, 39–43, 45–47). Two papers were not included in the data analyses, as the biomarkers they had assessed were insufficient for generating pooled results (38, 44). Among these studies, 11 assessed plasma MDA (30–35, 39, 41, 45–47), 6 papers measured plasma LP (29, 35–37, 39, 41), 9 studies looked for changes in TAC in plasma (30–32, 34, 39, 41, 43, 46, 47)3 trials assessed the plasma activity of GPx (33, 41, 42), and 3 of them looked for changes in plasma SOD activity (32, 41, 42). Available data on plasma F2-Isoprostanes (n=2) and Plasma 8-iso PGF-2α (n=2) were not sufficient for performing analyses. The flow diagram of the study screening and selection process is illustrated in **Figure 1**.

### 3.1 Characteristics of the included studies

The complete characteristics of 19 trials included in the current systematic review are available in **Table 1**. All the RCTs were published between the years 2000 and 2021. The studies were performed on three continents, including Asia (30–34, 36, 37, 45, 46), Europe (36, 38–40, 44, 47), and America (29, 35, 41–43). Six studies were carried out on males (32, 33, 36, 39, 40, 43) and five on female subjects (31, 35, 37, 45, 46) exclusively, and eight studies’ participants were both males and females (29, 30, 34, 38, 41, 42, 44, 47). The sample sizes of extracted trials varied from 14 to 256, building up a total sample size of 1253 (Intervention: n= 632, Control: n= 621) subjects in the systematic review and 965 (Intervention: n= 491, Control: n= 474) for the meta-analysis. Most studies were conducted on adult subjects; only a study by Neziroglu et al. in 2010 was on adolescents (36). The doses of vitamin C and E supplements varied from 250 to 1000 and 84 to 536 mg/d, respectively. The duration of intervention in the included studies was a value in the range of 2 to 144 weeks.

**Table 1:** Summary of randomized clinical trials on the effects of the combination of vitamin E and C on plasma oxidative stress and antioxidant capacity.

Author (year)	Location	Subjects, n	Health condition	Age^1^	Design	Dosage^6^	Duration^8^	Control	Outcome(s)^7^		
									Biomarker (Unit)	Intervention	Control
Aghdassi et al., 2003 (29)	Toronto, Canada	Int: 24 Con: 26 M/F	CD	Int: 38.3 ± 15.34 Con: 36.5 ± 9.15	R, DB, P	VitE: 536 VitC: 1000	4	Placebo	Plasma LP (µmol/L)	-0.70 ± 0.88^Ϯ^	2.11 ± 5.35
Mier-Cabrera et al., 2007 (35)	Mexico City, Mexico	Int: 16 Con: 18 F	Endometriosis	Int: 32.7 ± 2.36 Con: 32.8 ± 2.49	R, DB, P	VitE: 84 VitC: 343	24	A similar bar with no supplement included	Plasma MDA (µmol/L)	-16.58 ± 0.98^Ϯ^	-3.86 ± 1.66
									Plasma LP (µmol/L)	-6.15 ± 1.03^Ϯ^	-1.46 ± 1.00
Paulsen et al., 2014 (38)	Norway	Int: 17 Con: 17 M/F	Healthy adults, endurance training	Int: 25 ± 5 Con: 24 ± 6	R, DB, P	VitE: 235 VitC: 1000	11	Pills with the same shape and appearance	Plasma 8-iso PGF-2α (pmol/L)	-4.54 ± 2.87	40.3 ± 2.87
Plantinga et al., 2007 (39)	Italy	Int: 30 Con: 30 M	Essential hypertension	50	R, DB, CO	VitE: 268 VitC: 1000	8	Placebo	Plasma MDA (µmol/L)	-0.3 ± 0.22^Ϯ^	0.02 ± 0.19
									Plasma LP (µmol/L)	-1.06 ± 1.09	-0.02 ± 1.01
									Plasma TAC (mmol/L)	0.042 ± 0.001^Ϯ^	-0.006 ± 0.001
Rodrigo et al., 2008 (43)	Santiago, Chile	Int: 55 Con: 55 M	Essential hypertension	46	R, DB, P	VitE: 268 VitC: 1000	8	Indistinguishable capsules	Plasma TAC (mmol/L)	0.137± 0.047^Ϯ^	0.003± 0.068
									Plasma 8-iso PGF-2α (pmol/L)	-17.08 ± 8.67	1.46 ± 6.86
Ullegaddi et al., 2006 (47)	Sheffield, UK	Int (VitE+C): 24 Con: 24 M/F	After acute ischemic stroke	Int: 75± 10.24 Con: 78.67 ± 8.67	R, SB, P	VitE: 536 VitC: 500	2	No placebo	Plasma MDA (µmol/L)	-0.07 ± 0.21^Ϯ^	0.07 ± 0.22
									Plasma TAC (mmol/L)	0.07 ± 0.08^Ϯ^	-0.06 ± 0.23
		Int (Vit E+C+B): 24 Con (VitB): 24 M/F		Int: 74.67 ± 12.61 Con: 75.33 ± 10.24					Plasma MDA (µmol/L)	-0.03 ± 0.20	-0.01 ± 0.17
									Plasma TAC (mmol/L)	0.00 ± 0.09	0.01 ± 0.15
Alavi-Naeini et al., 2013 (30)	Isfahan, Iran	Int: 127 Con: 129 M/F	Elderly with mild cognitive impairment	Int: 66.50 ± 4.39 Con: 66.3 ± 4.31	R, SB, P	VitE: 300 VitC: 400	12	Placebo with an identical condition	Plasma MDA (µmol/L)	0.21 ± 0.20^Ϯ^	0.61 ± 0.45
									Plasma TAC (mmol/L)	0.70 ± 0.30^Ϯ^	0.43 ± 0.35
Amini et al., 2021 (31)	Tehran, Iran	Int: 30 Con: 30 F	Endometriosis, pelvic pain	Int: 35.7 ± 5.71 Con: 38.03 ± 6.47	R, TB, P	VitE: 536 VitC: 1000	8	Placebo	Plasma MDA µmol/L)	-23.26 ± 14.35^Ϯ^	2.42 ± 7.75
									Plasma TAC (mmol/L)	0.01 ± 0.21	0.08 ± 0.16
El-Aal et al., 2018 (33)	Gaza, Palestine	Int: 10 Con: 10 M	T2DB, metformin treatment	51.02 ± 5.44	R, SB, P	VitE: 268 VitC: 500	12	Placebo	Plasma MDA µmol/L)	0.21 ± 0.37^Ϯ^	1.27 ± 1.03
									Plasma GPx (U/L)	207.07 ± 55.43^Ϯ^	70.44 ± 41.11
Lai 2008 (34)	Taipei, Taiwan	Int (Cr+VitC+VitE): 10 Con (Cr): 10 M/F	T2DM	Int: 51.5 ± 1.7 Con: 53.2 ± 2.0	R, DB, P	VitE: 536 VitC: 1000	24	Chromium	Plasma TAC (mmol/L)	0.2 ± 0.2	0.13 ± 0.02
									Plasma MDA µmol/L)	-1.92 ± 0.04^Ϯ^	-1.01 ± 0.02
Neziroglu et al., 2010 1 (37)	Turkey	Int: 10 Con: 11 F	Fibromyalgia, physical activity	Int: 37.4 ± 4.0 Con: 37.8 ± 8.7	R, NB, P	VitE: 150 VitC: 500	12	No placebo	Plasma LP (µmol/L)	-1.5 ± 0.3	-1.4 ± 0.31
Neziroglu et al., 2010 2 (36)	Turkey	Int: 7 Con: 7 M	Maximal exercising basketball players	16.8 ± 1.06	R, SB, P	VitE: 150 VitC: 500	5	Placebo	Plasma LP (µmol/L)	0.35 ± 0.14^Ϯ^	0.74 ± 0.09
Porkkala-Sarataho et al., 2000 (40)	Finland	Int: 15 Con: 11 M	Nondepleted men	Int: 54.6 ± 6.7 Con: 55.0 ± 6.1	R, DB, P	VitE: 182 VitC: 500	144	Placebo	Plasma TRAP (µmol/L)	-139.9 ± 255.1	-128.6 ± 214.4
Tam et al., 2005 (46)	Hong Kong	Int: 20 Con: 19 F	Systemic lupus erythematosus	Int: 44 ± 6 Con: 48 ± 11	R, DB, P	VitE: 536 VitC: 500	48	Matched placebo	Plasma MDA µmol/L)	-0.10 ± 0.06	-0.07 ± 0.09
									Plasma TAC (mmol/L)	0.005 ± 0.0548^Ϯ^	-0.080 ± 0.127
Retana-Ugalde et al., 2009 (42)	Mexico City, Mexico	Int: 25 Con: 25 M/F	Healthy older adults	Int: 67 ± 7.5 Con: 65± 11	R, DB, P	VitE: 268 VitC: 1000	48	Matched placebo	Plasma LP (µmol/L)	-0.12 ± 0.02^Ϯ^	-0.09 ± 0.02
									Plasma SOD (U/L)	1.0 ± 1.2	3.0 ± 1.25
									Plasma GPx (U/L)	4204 ± 1291.95^Ϯ^	1425 ± 1496.18
Salonen et al., 2003 (44)	Denmark	Int: 58 Con: 64 M	Smoking and nonsmoking men	45-69	R, DB, P	VitE: 182 VitC: 500	144	Placebo identical in appearance, size, and color	Plasma F2-Isoprostanes (ng/L)	-2 ± 15.23	2.9 ± 15.2
		Int: 66 Con: 66 F	Postmenopausal women						Plasma F2-Isoprostanes (ng/L)	-2.9 ± 28.43	-4.0 ± 47.12
Retana-Ugalde et al., 2008 (41)	Mexico City, Mexico	Int_a_: 22 Int_b_: 22 Con: 22 M/F	Healthy elderly adults	Int_1_: 67 ± 28.14 Int_2_: 67 ± 28.14 Con: 66 ± 42.21	R, DB, P	Int_a_ VitE: 268 VitC: 500	24	Placebo with a pharmaceutical presentation similar to that of the treatment	Plasma MDA µmol/L)	-0.03 ± 0.004	-0.04 ± 0.004
									Plasma TAC (mmol/L)	0.05 ± 0.03^Ϯ^	-0.03 ± 0.07
									Plasma SOD (U/L)	5.9 ± 4.23	5.6 ± 3.31
									Plasma GPx (U/L)	1905 ± 1275.68^Ϯ^	941 ± 484.12
						Int_b_ VitE: 268 VitC: 1000			Plasma MDA µmol/L)	-0.05 ± 0.008^Ϯ^	-0.04 ± 0.004
									Plasma TAC (mmol/L)	0.09 ± 0.26^Ϯ^	-0.03 ± 0.07
									Plasma SOD (U/L)	5.6 ± 4.06	5.6 ± 3.31
									Plasma GPx (U/L)	2258 ± 493.77^Ϯ^	941 ± 484.12
Bagheri-Hosseinabadi et al., 2020 (32)	Semnan, Iran	Int: 21 Con: 19 M	Power plant workers	Int, Con: 20-50 P-value=0.457	R, DB, P	VitE: 268 VitC: 1000	12	Cocoa milk mixed (Int) or not mixed (Con) with supplements	Plasma MDA (µmol/L)	-3.10 ± 3.02^Ϯ^	-0.86 ± 2.46
									Plasma TAC (mmol/L)	0.54 ± 0.34^Ϯ^	-0.03 ± 1.07
									Plasma SOD (U/L)	80.89 ± 1.08^Ϯ^	12.45 ± 1.03
Taghiyar et al., 2013 (45)	Isfahan, Iran	Int: 14 Con: 15 F	Athletes	Int: 33.9 ± 5.61 Con: 38.1 ± 5.42	R, DB, P	VitE: 268 VitC: 250	4	Placebo manufactured in the same company as supplements	Plasma MDA (µmol/L)	-2.7 ± 0.47	-3.4 ± 1.36

^1^Mean ± SD or range (years), ^2^µmol/L, ^3^mmol/L, ^4^mg/d, ^5^µg/d, ^6^mg/day, ^7^Mean ± SD, ^8^weeks, ^Ϯ^significantly different mean difference compared to control. All the data is presented as Mean ± SD (standard deviation).

Int, intervention; Con, control; M, male; F, female; MDA; malondialdehyde NR, not reported; CD, Crohn’s Disease; SBD, small bowel disease; SBR, small bowel restriction; R, randomized; SB, single-blind; DB, double-blind; TB, triple-blind; NB, no blinding; P, parallel; CO, crossover; NO, no medications; NS, no supplements; TC, total cholesterol; BP, blood pressure; EL, educational levels; LSVE, lipid standardized vitamin E; PA, physical activity; TAC, total antioxidant capacity; LP, lipid peroxides; TRAP, total peroxyl radical–trapping antioxidant biomarker; GPx, glutathione peroxidase.

Among all included studies, only one study had a crossover design (39), while all the remaining studies employed a paralleled design. Regarding anonymizing, only one study lacked any degree of blindness (37), while, 4 studies were single-blinded (30, 33, 36, 47), 13 trials were double-blinded (29, 32, 34, 35, 38–46), and one was triple-blinded (31). In five studies, healthy subjects were intervened (32, 36, 38, 41, 42, 45) and the other studies were conducted on unhealthy cases. None of the included could be labeled low-risk as all the studies were at least one high-risk or unclear-risk domain. Five studies had an unclear risk of bias for being unclear in at least one domain of RoB2 (32, 34, 39, 43, 46). The remaining thirteen studies all had a high risk of bias for being high-risk in at least one domain of RoB2 (29–33, 35–38, 40–42, 44, 45, 47). Based on the scores obtained from Rob2, none of the included studies were labeled with Poor quality. Two studies were considered with Adequate quality (36, 37). Eleven studies’ qualities were Good (29–31, 33, 38, 47). Finally, six studies had an Excellent quality (32, 34, 35, 40, 46, 47) (see **Table 2**).

**Table 2:** Results of risk of bias assessment for randomized clinical trials included in the current meta-analysis on the effects of vitamin E and C co-supplementation on oxidative stress and plasma antioxidant capacity biomarkers^1^.

Study	Random sequence generation	Allocation concealment	Selective reporting	Blinding (participants and personnel)	Blinding (outcome assessment)	Incomplete outcome data	Low adherence to intervention2	Results		
								Overall risk of bias	Given score^3^	Overall quality^4^
Aghdassi et al., 2003 (29)	L	L	H	L	U	H	L	H	2	Good
Mier-Cabrera et al., 2007 (35)	L	L	H	L	U	L	L	H	4	Excellent
Paulsen et al., 2014 (38)	L	U	H	L	U	U	U	H	1	Good
Plantinga et al., 2007 (39)	L	U	L	L	U	U	U	U	3	Good
Rodrigo et al., 2008 (43)	L	U	L	L	U	L	U	U	4	Excellent
Ullegaddi et al., 2006 (47)	L	L	L	H	L	U	U	H	3	Good
Alavi-Naeini et al., 2013 (30)	L	L	L	H	U	U	U	H	2	Good
Amini et al., 2021 (31)	L	L	H	L	L	U	U	H	3	Good
El-Aal et al., 2018 (33)	L	U	L	H	H	L	U	H	1	Good
Lai 2008 (34)	L	U	L	L	U	L	U	U	4	Excellent
Neziroglu et al., 2010 1 (37)	L	U	H	H	H	L	U	H	-1	Adequate
Neziroglu et al., 2010 2 (36)	L	U	H	H	H	L	U	H	-1	Adequate
Porkkala-Sarataho et al., 2000 (40)	L	L	H	L	U	L	L	H	4	Excellent
Tam et al., 2005 (46)	L	L	L	L	U	L	L	U	6	Excellent
Retana-Ugalde et al., 2009 (42)	L	U	H	L	U	L	U	H	2	Good
Salonen et al., 2003 (44)	L	L	H	L	U	H	U	H	1	Good
Retana-Ugalde et al., 2008 (41)	L	U	H	L	U	L	U	H	2	Good
Bagheri-Hosseinabadi et al., 2020 (32)	L	L	L	L	U	L	U	U	5	Excellent
Taghiyar et al., 2013 (45)	L	L	H	L	U	L	U	H	3	Good

^1^Each study was assessed for risk of bias using the Cochrane Risk of Bias Assessment tool version 2 (RoB2) (12). Domains of assessment included random sequence generation, allocation concealment, reporting bias, performance bias, detection bias, attrition bias, and other sources of bias (insufficient compliance to treatment). Each domain was scored as H (high risk) if it contained methodological flaws that may have affected the results, L (low risk), if the flaw was deemed inconsequential, and U (unclear risk) if information wasn’t sufficient to determine.

^2^Compliance of 80 percent or more was considered a low risk.

^3^We calculated an overall quality score for each study by considering -1, 0, and 1 value for H, U, and L risk of bios respectively, and summing them up.

^4^Poor: -7 to -3.5, Adequate: -3.4 to 0, Good: 0.1 to 3.5, Excellent: 3.6 to 7.

### 3.2 Findings from the systematic review

Among 10 studies that assessed changes in plasma concentration of MDA, 7 reported a significant reduction in plasma MDA following supplementation (30–35, 39), whereas 4 others failed to show a significant effect (41, 45–47). Four trials showed a significant reducing effect on plasma LP concentrations for co-supplementation with vitamins E and C (29, 35, 36, 42); in contrast, the other two did not show the same result and their results were insignificant (37, 39). Out of 9 studies assessing plasma TAC, 7 reported a significant positive effect for the intervention compared to the control (30, 32, 39, 41, 43, 46, 47); whereas the results of two of the papers were insignificant (31, 34). Of three studies assessing plasma GPx activity, only in the study by Bagheri-Hosseinabadi et al. supplementation was significantly effective in elevating plasma GPx activity compared to the control (32), while one study showed a significant negative effect for the intervention compared to the control (42) and another study showed an insignificant increase in co-supplementation (41). All three studies in which SOD activity of plasma was assessed, converged the significant effectiveness of vitamins E and C co-supplementation for the elevation of this biomarker (33, 41, 42). Two studies assessed plasma 8-iso PGF-2α and both reported a significant drop in the biomarker following supplementation (38, 43). The trial by Salonen et al., in which plasma F2-Isoprostanes were assessed as a marker of oxidative stress, found no significant effect in either men or women [Overall effect: -4.063 (-9.042, 0.916) p-value = 0.110] (44).

### 3.3 Findings from the meta-analysis

A total of 17 trials were included in the meta-analysis building a total sample size of 965 (29–37, 39–43, 45–47).

#### 3.3.1 The effect of vitamins E and C on plasma Malonaldehyde concentrations

**Figure 2:**
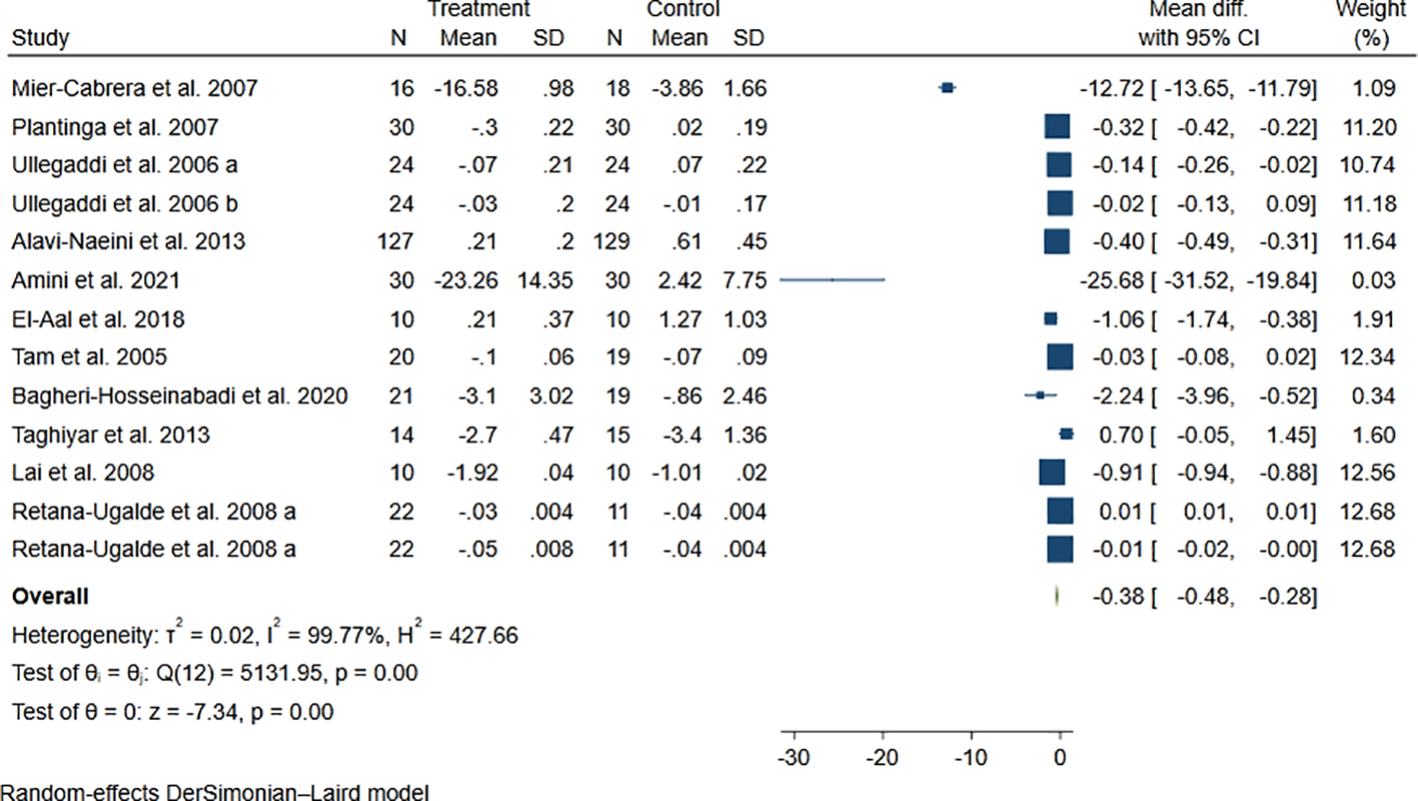
The forest plot of the meta-analysis on the effect of vitamins E and C on plasma concentration of MDA compared to control. Horizontal lines represent 95% CIs. Diamonds represent pooled estimates from random-effects analysis. CI, confidence interval.

The analysis was based on 11 trials with a total sample size of 720 subjects (30–35, 39, 41, 45–47). Supplementation with vitamins E and C concurrently was found significantly effective in reducing the plasma concentration of MDA compared to the control based on the combination of 13 effect sizes [weighted mean difference (WMD): -0.38, 95% confidence interval (CI): -0.48, -0.28 µg/L, P <0.001] (see **Figure 2**).

However, evidence of a high heterogeneity was observed between studies (*I^2^ =* 99.97%, P < 0.001). We carried out subgroup analyses and meta-regression tests in order to detect the potential source(s) of heterogeneity (see **Tables 3**, **4**). The application or lack of a placebo in the control groups, duration of intervention, and dosage of vitamin E explained this high between-study heterogeneity. According to the subgroup analyses, studies with a placebo administration showed a significant reduction in MDA, unlike those without one. Additionally, results for unhealthy subjects were substantial, but not in a healthy state. Based on meta-regression tests, an increase in duration of intervention may higher the potential beneficial effect of these supplements on MDA [Coefficient: -0.052, 95% CI: -0.093, 0.012 µg/L, P = 0.012]. Moreover, an increase in dosage of vitamin E resulted in a significant positive coefficient in meta-regression test [Coefficient: 0.013, 95% CI: 0.010, 0.017 µg/L, P < 0.001].

**Table 3:** Subgroup analyses for the effects of vitamin E and C co-supplementation on plasma oxidative stress and antioxidant capacity.

Subgroup	Effect size, n	WMD (95% CI)^1^	P-within^2^	I^2^ (%)^3^	P-heterogeneity^4^
Vitamin E and C co-supplementation on plasma MDA concentrations					
Overall	13	-0.38 (-0.48, -0.28)	<0.001	99.8	<0.001
Intervention duration (week)					
<8	5	-0.15 (-0.55, 0.24)	0.45	95.8	<0.001
8-24	5	-3.18 (-4.03, -2.34)	<0.001	99.5	<0.001
>24	3	-0.00 (-0.02, 0.02)	0.73	47.5	<0.001
Study location					
Asia	7	-0.68 (-1.19, -0.16)	0.01	99.5	<0.001
Europe	3	-0.16 (-0.34, 0.02)	0.08	87.5	<0.001
America	3	-0.09 (-0.17, -0.01)	0.02	99.7	<0.001
Control					
Placebo	11	-0.47 (-0.58, -0.35)	<0.001	99.8	<0.001
No placebo	2	-0.08 (-0.19, 0.04)	0.20	53.3	0.14
Health condition					
Healthy	4	0.00 (-0.02, 0.02)	0.98	94.6	<0.001
Unhealthy	9	-1.62 (-2.08, -1.15)	<0.001	99.6	<0.001
Blinding					
Single-blinded	4	-0.27 (-0.51, -0.02)	0.03	92.2	<0.001
Double-blinded	8	-0.44 (-0.57, -0.32)	<0.001	99.9	<0.001
Triple-blinded	1	-25.68 (-31.52, -19.84)	<0.001	0	0
Study quality					
Good	9	-0.09 (-0.13, -0.05)	<0.001	96.9	<0.001
Excellent	4	-3.56 (-4.43, -2.68)	<0.001	99.8	<0.001
Vitamin E and C co-supplementation on plasma LP concentrations					
Overall	6	-1.01 (-1.49, -0.54)	<0.001	96.9	<0.001
Intervention duration (week)					
<8	3	-0.43 (-1.07, 0.21)	0.19	71.7	0.029
8-24	2	-2.38 (-6.88, 2.11)	0.30	99.3	<0.001
>24	1	-0.41 (-0.45, -0.37)	<0.001	0	0
Study location					
Asia	2	-0.27 (-0.55, 0.01)	0.06	74.2	0.049
Europe	1	0.00 (-0.55, 0.55)	1.00	0	0
America	3	-2.62 (-5.98, 0.74)	0.13	98.7	<0.001
Control					
Placebo	5	-1.28 (-1.85, -0.72)	<0.001	97.5	<0.001
No placebo	1	-0.10 (-0.36, 0.16)	0.45	0	0
Health condition					
Healthy	2	-0.41 (-0.45, -0.37)	<0.001	0.0	0.762
Unhealthy	4	-1.84 (-4.00, 0.32)	0.10	98.1	<0.001
Blinding					
Non-blinded	1	-0.10 (-0.36, 0.16)	0.45	0	0
Single-blinded	1	-0.39 (-0.51, -0.27)	<0.001	0	0
Double-blinded	4	-1.91 (-3.84, 0.01)	0.05	98.1	<0.001
Study quality					
Adequate	2	-0.27 (-0.55, 0.01)	0.06	74.2	0.049
Good	3	-0.44 (-1.08, 0.19)	0.17	72.2	0.027
Excellent	1	-4.69 (-5.37, -4.01)	<0.001	0	0
Vitamin E and C co-supplementation on plasma TAC					
Overall	11	0.09 (0.05, 0.13)	<0.001	90.9	<0.001
Intervention duration (week)					
<8	5	0.05 (-0.01, 0.11)	0.08	94.4	<0.001
8-24	3	0.22 (0.03, 0.41)	0.02	77.6	0.011
>24	3	0.09 (0.05, 0.12)	<0.001	0.0	0.819
Study location					
Asia	5	0.12 (-0.02, 0.26)	0.09	88.1	<0.001
Europe	3	0.05 (-0.01, 0.10)	0.08	62.7	0.069
America	3	0.11 (0.07, 0.15)	<0.001	58.1	0.092
Control					
Placebo	9	0.10 (0.05, 0.15)	<0.001	92.3	<0.001
No placebo	2	0.06 (-0.08, 0.19)	0.43	80.9	0.022
Health condition					
Healthy	3	0.11 (0.01, 0.21)	0.05	49.4	0.138
Unhealthy	8	0.09 (0.03, 0.14)	0.01	93.1	<0.001
Blinding					
Single-blinded	3	0.13 (-0.04, 0.30)	0.14	92.5	<0.001
Double-blinded	7	0.09 (0.04, 0.14)	<0.001	91.3	<0.001
Triple-blinded	1	-0.07 (-0.16, 0.02)	0.15	0	0
Study quality					
Good	7	0.08 (0.02, 0.13)	0.01	86.6	<0.001
Excellent	4	0.11 (0.06, 0.17)	<0.001	49.5	0.114

WMD, weighted mean difference; CI, confidence interval; MDA, malondialdehyde; TAC, total antioxidant capacity; LP, lipid peroxides; OS, oxidative stress.

^1^Obtained from the random-effects model.

^2^Refers to the mean (95% CI).

^3^Inconsistency, percentage of variation across studies due to heterogeneity.

^4^Obtained from the Q-test.

**Table 4:** Findings of the meta-regression on the effect of vitamin E and C co-supplementation on plasma oxidative stress and antioxidant capacity.

Factor	Effect size, n	Coefficient (95% CI)^1^	P-value
Vitamin E and C co-supplementation on plasma MDA concentrations			
Dosage			
Vitamin E^3^	13	0.0133 (0.010, 0.016)	<0.001
Vitamin C^3^	13	0.001 (-0.001, 0.003)	0.272
Duration of intervention^2^	13	-0.052 (-0.934, -0.012)	0.012
Quality score	13	-0.398 (-0.819, 0.023)	0.064
Vitamin E and C co-supplementation on plasma LP concentrations			
Dosage			
Vitamin E^3^	6	0.000 (-0.012, 0.013)	0.966
Vitamin C^3^	6	0.002 (-0.003, 0.008)	0.413
Duration of intervention^2^	6	-0.006 (-0.117, 0.105)	0.917
Quality score	6	-0.550 (-1.563, 0.463)	0.206
Vitamin E and C co-supplementation on plasma TAC concentrations			
Dosage			
Vitamin E^3^	11	0.000 (-0.001, 0.000)	0.109
Vitamin C^3^	11	0.000 (0.000, 0.000)	0.422
Duration of intervention^2^	11	0.001 (-0.004, 0.005)	0.816
Quality score	11	-0.004 (-0.026, -0.061)	0.883
Vitamin E and C co-supplementation on plasma GPx concentrations			
Dosage			
Vitamin C^3^	4	2.926 (-0.301, 6.154)	0.076
Duration of intervention^2^	4	78.438 (58.660, 98.217)	<0.001
Quality score	4	1516.067 (-2874.898, 5907.031)	0.276
Vitamin E and C co-supplementation on plasma SOD concentrations			
Dosage			
Vitamin C^3^	4	0.044 (-0.138, 0.225)	0.637
Duration of intervention^2^	4	-1.564 (-3.875, 0.747)	0.185
Quality score	4	23.142 (21.092, 25.191)	<0.001

CI, confidence interval; MDA, malondialdehyde; TAC, total antioxidant capacity; LP, lipid peroxides; OS, oxidative stress; GPx, glutathione peroxidase; SOD, superoxide dismutase.

^1^Obtained from the random-effects model.

^2^weeks.

^3^mg/day.

The overall estimate for the effect of vitamins E and C co-supplementation on MDA was not affected significantly by excluding every single study in the sensitivity analysis (range of summary estimates: -0.48, -0.28). Moreover, no evidence of publication bias was observed based on Egger’s test (P = 0.76).

#### 3.3.2 The effect of vitamins E and C on plasma lipid peroxides concentrations

**Figure 3:**
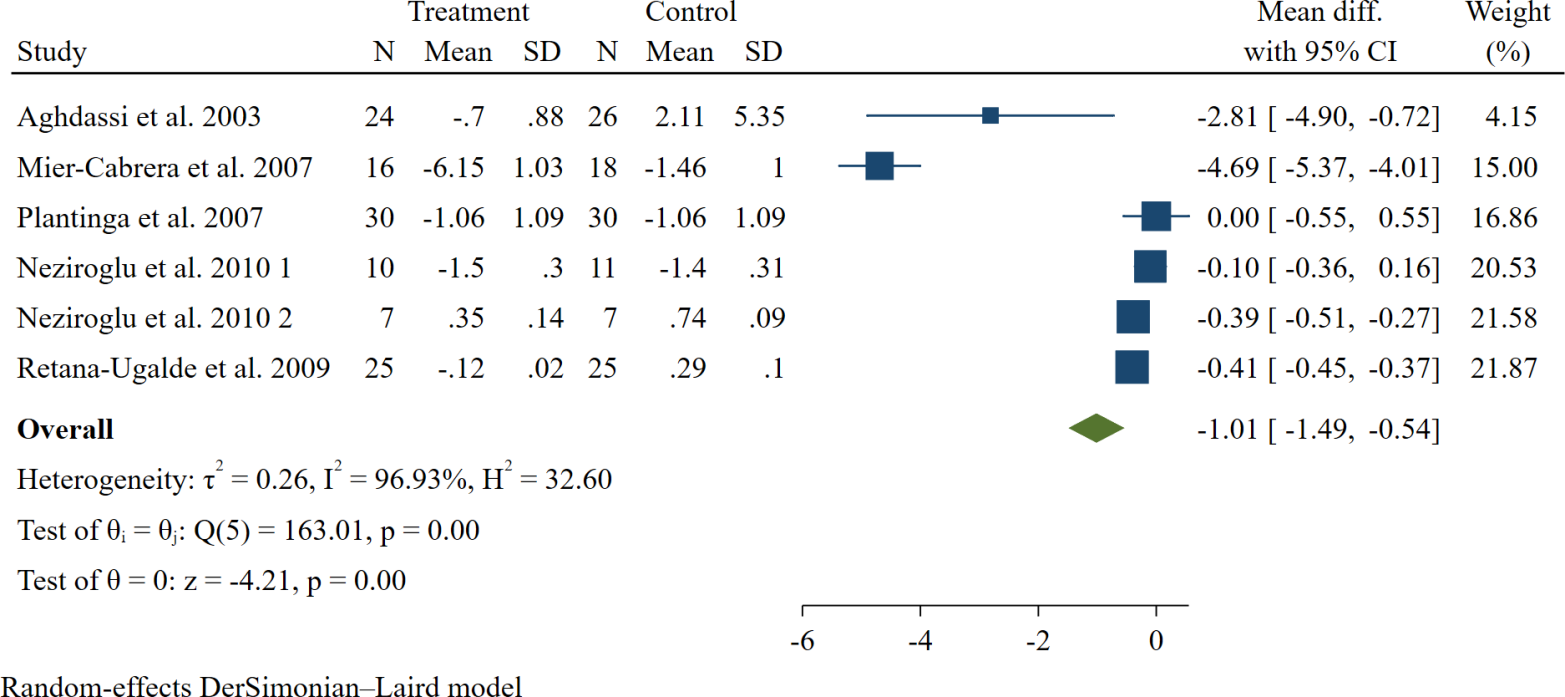
The forest plot of the meta-analysis on the effect of vitamins E and C on plasma concentration of plasma LP compared to control. Horizontal lines represent 95% CIs. Diamonds represent pooled estimates from random-effects analysis. CI, confidence interval.

Overall, 6 studies with 6 arms were included in the analysis, building up a sample size of 229 subjects (29, 35–37, 39, 42). According to the analysis, co-supplementation significantly reduced the plasma concentration of LP [WMD: -1.01, 95% CI: -1.49, -0.54 µg/L, P < 0.001] (see **Figure 3**).

Similar to MDA, the meta-analysis on this plasma biomarker showed a high between-study heterogeneity (*I^2^ =* 96.93%, P < 0.001), and the same tests were taken into account to detect the possible sources (see **Tables 3**, **4**). Based on results from subgroup analysis, study quality could be the potential source of heterogeneity. Once again, only the analysis on the papers with a placebo showed a significant effect, not the one without it. Excluding every single study did not affect the results significantly for LP (range of summary estimates: -1.49, -0.54). No evidence of publication bias was observed in Egger’s test (P = 0.414).

#### 3.3.3 The effect of vitamins E and C on plasma total antioxidant capacity

**Figure 4:**
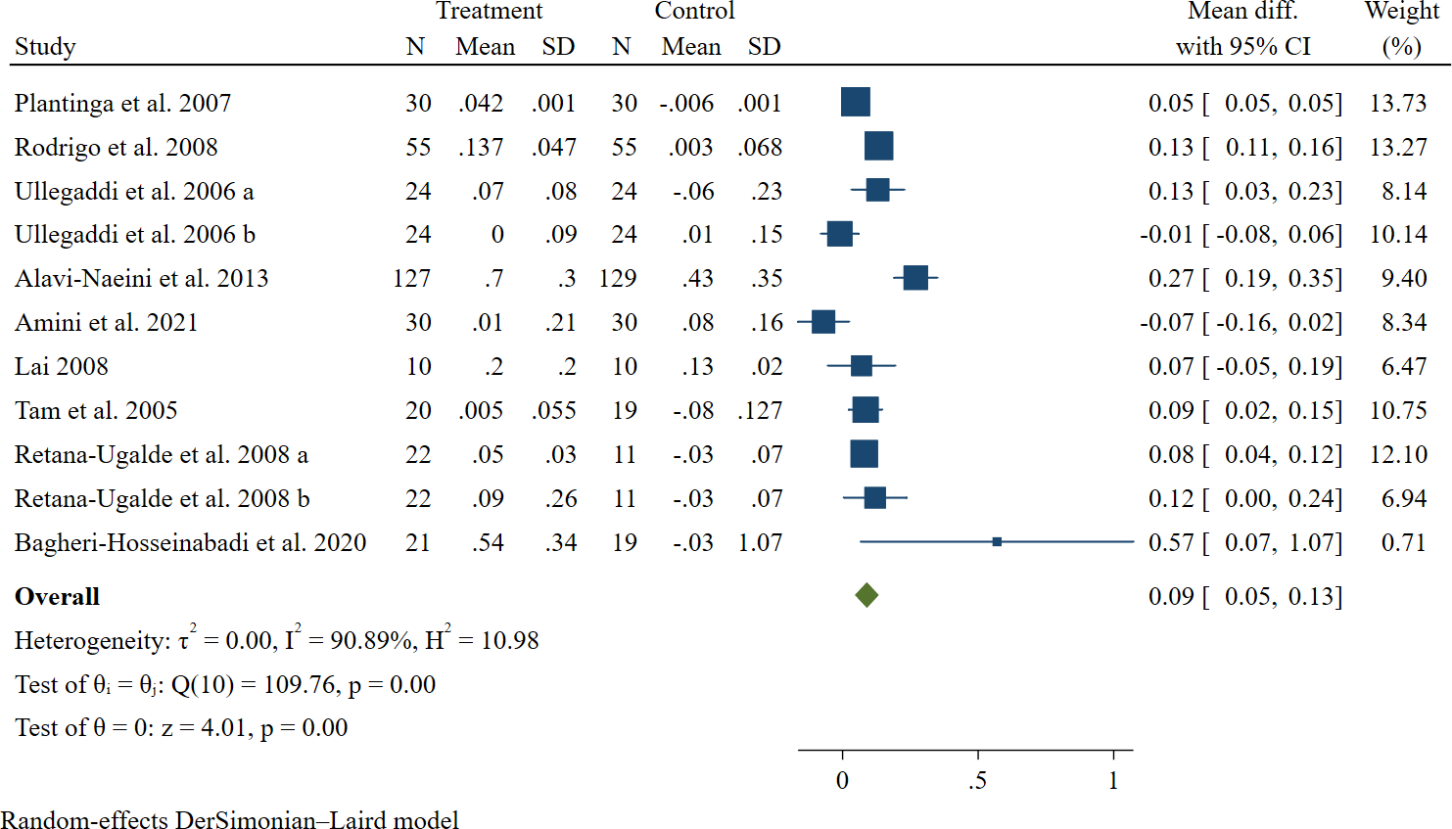
The forest plot of the meta-analysis on the effect of vitamins E and C on plasma concentration of TAC. Horizontal lines represent 95% CIs. Diamonds represent pooled estimates from random-effects analysis. WMD: CI, confidence interval.

In regards to plasma TAC, 9 studies with 11 effect sizes were considered eligible for inclusion in the analyses in which 747 people were included (30–32, 34, 39, 41, 43, 46, 47). Co-supplementation with vitamins E and C significantly elevated plasma TAC according to our results based on random-effect analysis [WMD: 0.09, 95% CI: 0.05, 0.13 mmol/L, P <0.001] (see **Figure 4**).

However, again there was evidence of high heterogeneity between studies and the same analyses were performed to find the possible sources of this heterogeneity (*I^2^ =* 90.89%, P < 0.001) (see **Tables 3**, **4**). According to the subgroup analyses, differences in study location, the health condition of participants, and study quality are the potential sources of heterogeneity. No significant change was observed by excluding every single study in the sensitivity analysis (range of summary estimates: 0.046, 0.133). There was no evidence of publication bias based on Egger’s test (P = 0.110).

#### 3.3.4 The effect of vitamins E and C on plasma glutathione peroxidase activity

With 3 included studies, a total of 136 participants were included based on 4 intervention groups. The analysis showed a significant positive effect on the activity of plasma GPx following the co-supplementation [WMD: 1251.74, 95% CI: 258.92, 2244.56 U/L, P = 0.013] (see **Supplementary Figure S1**).

Regarding heterogeneity, a high level was observed (*I^2^* = 96.78%, P<0.001). The potential source was found probably to be the duration of studies according to meta-regression tests [Coefficient: 78.44, 95% CI: 58.66, 98.22 U/L, P < 0.001] (see **Table 4**). According to sensitivity analysis by excluding the Retana-Ugalde et al. study in 2008 or the study by El-Al, results significantly change in the meta-analyses. However, there was no possibility of publication bias based on Egger’s test (P =0.060).

#### 3.3.5 The effect of vitamins E and C on plasma superoxide dismutase activity

Only 3 studies measured plasma SOD as a biomarker for antioxidant status which resulted in 156 subjects in 4 intervention arms (32, 41, 42). The pooled analysis failed to find a significant effect of vitamin E and C on plasma SOD [WMD: 16.69, 95% CI: -29.40, 62.78 U/L, P = 0.278] (see **Supplementary Figure S2**).

There was also evidence of extremely high between-study heterogeneity (*I^2^* = 99.99%, P<0.001) which was explained by the duration of interventions based on meta-regression tests. According to meta-regression, there is a linear relationship between the study quality and the effectiveness of the supplementation [Coefficient: 23.14, 95% CI: 24.09, 25.19 U/L, P < 0.001] (see **Table 4**), and this was considered as the potential source of heterogeneity. No significant change was observed by excluding every single study in the sensitivity analysis (range of summary estimates: -29.40, 62.78). No evidence of publication bias was visible through Egger’s test (P = 0.740).

#### 3.3.6 Findings from GRADE assessment

Based on the GRADE assessment tool, the overall quality of evidence was measured for meta-analysis of each biomarker. The quality of evidence was high for MDA, LP, and GPx; the quality was regarded as moderate for TAC and it was low for SOD (see **Table 5**). Optimal sample size (OIS) was only met for GPx analysis. The studies by Ullegaddi et al., Amini et al., Neziroglu et al., and Tam et al. most contributed in this insufficiency (31, 36, 46, 47). However, sensitivity analyses did not show significant change in results after excluding these studies.

**Table 5:** Findings from GRADE assessment on the effect of vitamin E and C co-supplementation on plasma oxidative stress and antioxidant capacity.

Certainty assessment							No of patients		Effect	Certainty	Importance
No of studies	Study design	Risk of bias	Inconsistency	Indirectness	Imprecision	Other considerations	Vitamin E and C	control	Absolute (95% CI)		
Malondialdehyde											
13	randomised trials	serious^a^	not serious^c^	not serious	serious^d^	strong association all plausible residual confounding would reduce the demonstrated effect	316	318	MD 0.38 µg/L lower (0.48 lower to 0.28 lower)	⊕⊕⊕⊕ High^a,c,d^	CRITICAL
Lipid peroxides											
6	randomised trials	serious^a^	not serious^c^	not serious	serious^d^	strong association all plausible residual confounding would reduce the demonstrated effect	112	117	MD 1.01 µg/L lower (1.49 lower to 0.54 lower)	⊕⊕⊕⊕ High^a,c,d^	IMPORTANT
Total antioxidant capacity											
11	randomised trials	serious^a^	not serious^c^	not serious	serious^d^	all plausible residual confounding would reduce the demonstrated effect	385	362	MD 0.09 mmol/L higher (0.05 higher to 0.13 higher)	⊕⊕⊕◯ Moderate^a,c,d^	CRITICAL
Glutathione peroxidase											
4	randomised trials	very serious^b^	not serious^e^	not serious	not serious	very strong association all plausible residual confounding would reduce the demonstrated effect	79	57	MD 1251.74 U/L higher (258.92 higher to 2244.56 higher)	⊕⊕⊕⊕ High^b,e^	IMPORTANT
Superoxide dismutase											
4	randomised trials	serious^a^	not serious^e^	not serious	very serious^d,f^	all plausible residual confounding would reduce the demonstrated effect	90	66	MD 16.69 U/L higher (29.4 lower to 62.78 higher)	⊕⊕◯◯ Low^a,e,d,f^	IMPORTANT

CI, confidence interval; MD, mean difference.

Explanations:

a. More than 20% of RCTs for this outcome had a high risk of bias for at least one component of the Cochrane risk of bias tool. Those biases did not have a significant effect on the results of RCTs.

b. More than 20% of RCTs for this outcome had a high risk of bias for at least one component of the Cochrane risk of bias tool. Those biases had a significant effect on the results of RCTs.

c. The I2 value was >50%, however, the high heterogeneity was explained in the subgroup analyses.

d. The sample size did not meet the optimal sample size (OIS).

e. The I2 value was >50%, however, the high heterogeneity was explained in the meta-regression tests.

f. The overall results were insignificant.

## 4 Discussion

The current meta-analysis showed a significant effect on reducing plasma MDA and LP (as biomarkers for oxidative stress) following the co-supplementation of vitamins E and C. Additionally, significant positive results were obtained from the analyses of plasma TAC and GPx activity. However, analysis of plasma SOD activity failed to show a significant overall result, which may be because of the small overall effect size for the analysis of this biomarker. According to the analyses, this intervention can reduce plasma MDA to some extent. Moreover, it might significantly reduce plasma LP by about 1.01µg/L and increase TAC to around 0.09 mmol/L. Although the results for the effect of the co-supplementation of vitamin C and E on serum GPx were statistically significant and included studies assessing this marker were high-quality, results in this regard should be interpreted cautiously, considering the small sample for this biomarker.

Vitamin C is a water-soluble nutrient that neutralizes aqueous peroxyl radicals and restores alpha-tocopherol as a fat-soluble oxidant detoxifier (29). Humans, like many other species, are unable to synthesize vitamin C as a result of a lack of a lactonase identified as SMP30 or regucalcin, which converts l-gluconate to l-gluconolactone (30). Vitamin C can be received adequately through a healthy daily diet to meet the recommended dietary allowance (RDA) in both sexes without the need for supplement consumption in a normal state. However, in some conditions, such as smoking and an oxidative state, increasing its intake through supplementation becomes necessary (31). Vitamin E is a fat-soluble vitamin, acting as a chain-breaking oxidant neutralizer, exclusively active in the fat-soluble phase in the body (48, 49). As explained earlier, ascorbic acid is essential for the restoration of alpha-tocopherol, the active form of vitamin E, from the oxidized form (alpha-tocopherolquinone) (8, 9). This vitamin can be absorbed from the diet in eight forms: alpha-, beta-, delta-, and gamma-tocopherols and tocotrienols (49). Most studies are focused on alpha-tocopherol, and other forms, like gamma-tocopherol, have not been fully explored (50). Alpha-tocopherol in its prime form, can be received through the consumption of vegetable oils (51). However, it is believed that more than 90 percent of Americans do not receive sufficient alpha-tocopherol to meet the estimated average requirement (EAR) for this nutrient (52). As explained earlier, vitamin E is essential for the maintenance of antioxidant capacity, similar to ascorbic acid except in lipidic phases (53, 54). Both of these antioxidants neutralize oxidants, such as ROS, by giving them their electron (reducing them) and converting them into less aggressive molecules (10, 11). However, there is no conclusion in this area, not all RCTs report the same result for the co-supplementation of vitamins E and C on oxidative stress and oxidant capacity, and the findings are incontinence. The study by Aghdassi et al. in 2013 showed that supplementation with 800 IU of vitamin E and 1000 mg of vitamin C reduces oxidative stress significantly (29). The trial by Amini et all which was published in 2021, showed the same results, and plasma MDA and ROS concentrations significantly declined following 800 IU of vitamin E and 1000 mg of vitamin C (31). Additionally, El-Al et al. found that this intervention significantly decreased levels of ROS and oxidative stress and elevated the antioxidant capacity of power plant workers (33). However, some trials failed to find a significant result. For instance, Taghiyar et al. found no significant effect of the co-supplementation on plasma MDA. All the participants of this study were female athletes with a normal level of MDA in plasma, and this might be the reason for these insignificant results (45).

Among the included studies, only four assessed the dietary intake of antioxidants (29, 30, 34, 40); two of them only dietary vitamin E and C (34, 40). Other studies did not assess this critical variable, which may alter the results in addition to the potential heterogeneity this may cause (as a result of not considering differences in baseline dietary antioxidant intake), hence, results in the current meta-analyses should be interpreted with caution.

As mentioned earlier, we found significant results for plasma MDA, LP, TAC, and GPx but not for the activity of plasma SOD. This insignificancy may be because of low power of analysis on included studies assessing SOD as them being too few and extremely heterogenous; and to have a more precise and conclusive result, more studies in this regard might be needed. According to the subgroup analyses, the effect of this co-supplementation may be more intense on MDA reduction when the degree of blindness is increased. Moreover, LP is reduced more when a placebo is administered for the control group based on the subgroup analyses. When the duration of intervention was less than eight weeks, the analysis on TAC had no significant result; moreover, a duration between eight to twenty-eight weeks showed the strongest effect on elevating the capacity. Studies in which no placebo was administered failed to have a significant result compared to the ones that used a placebo for blindness in their control groups. The studies that had excellent quality based on RoB2, showed a more intensive effect for the co-supplementation on TAC. Meta-regression tests were unable to find any significant linear beneficial effect for dosage, duration, and quality on LP and TAC. However, according to the tests, the antioxidant effect of vitamin E and C co-supplementation is significantly increased when the duration of the intervention is lengthened. Moreover, when the duration of intervention is increased the supplementation with vitamin E and C may be more effective in lowering MDA concentration. Interestingly, higher doses of vitamin E may decrease the effect of the intervention on MDA reduction according to meta-regression. A placebo effect, which is triggered by conditioning or expect, may plays a role in various conditions (like pain, depression etc.) by neurobiological and psychological mechanisms. Evidence suggests that placebo interventions may be able to regulate neurotransmitter activity and improve one’s overall well-being (55). This might explain the more significant results in studies in which placebo was administered.

In the current review and meta-analysis, we gathered all available data for the determination of the effectiveness of vitamins E and C on oxidative stress and antioxidant status and found significant results for most biomarkers. However, our work had some limitations and should be kept in mind when interpreting the results. The majority of studies did not assess dietary antioxidants, which may alter the results. We could not find a significant result for the effect of these vitamins on the activity of plasma SOD which may be a result of the small sample size for this marker. Moreover, all of our primary analyses showed considerably high between-studies heterogeneity. Based on the subgroup analyses and meta-regression tests, the administration or lack of placebo, studies’ quality, the health condition of participants and the study location, durations of interventions, and again quality of studies were the potential sources of heterogeneity for MDA, LP, TAC, GPx, and SOD respectively. In addition to that dosage of vitamin E and the duration of intervention may be other sources of heterogeneity for MDA analysis. Moreover, all the included studies only examined the effect of α-tocopherol and ignored the other forms of vitamin E. Although the overall analyses were conducted on both healthy and unhealthy patients, we performed subgroup analyses by separating these two groups. As most studies did not report compliance for their intervention, we were unable to perform meta-regression and subgroup analyses based on this, and future trials should report the adherence of participants to the study. And finally, the included studies failed to meet the calculated OIS for most outcomes (OIS was only met for GPx analysis), hence results should be generalized to the population for other four outcomes.

Although most of our results suggested that supplementation with vitamin E and C may be beneficial on most biomarkers of oxidative stress and antioxidant status, there was high heterogeneity between studies and results should be causally translate into clinical or public health practice. According to our analyses, this intervention may be most beneficial when its duration is between 8 to 24 weeks. Additionally, different populations reacted differently to the intervention and this should be considered while translation into practice. As there was not enough variation between dosage, we could not perform dose response analysis and thus, no optimum dose was found in this regard.

Our review showed that co-supplementation of vitamins α-tocopherol and ascorbic acid can beneficially affect oxidative stress and antioxidant status, significantly reduce plasma levels of MDA and LP, and significantly increase TAC and GPx. Future studies should have larger sample sizes and more adjusted confounders. Additionally, they should also assess the daily dietary intake of antioxidants as an important confounder via dietary assessment methods (e.g., dietary recalls and records). Moreover, further studies should examine the effect of other types of vitamin E, other than α-tocopherol, in combination with ascorbic acid, on oxidative stress biomarkers and antioxidant status. Although mechanisms suggest it may be more effective for these vitamins to be supplemented together, it is not clear if this is true in practice; hence, a network meta-analysis in this regard (which compares vitamin E, vitamin C, or a combination of them as supplement on oxidative stress biomarkers) or Bayesian meta-analytic approaches would be a very interesting and critical topic for future studies.

## Data Availability

The original contributions presented in the study are included in the article/**Supplementary Material**. Further inquiries can be directed to the corresponding authors.
