# Supplementary material for: The effect of co-administration of vitamin E and C supplements on plasma oxidative stress biomarkers and antioxidant capacity: a GRADE-assessed systematic review and meta-analysis of randomized controlled trials with meta-regression
Source: Front Immunol. 2025 Jul 16;16:1547888. doi: 10.3389/fimmu.2025.1547888 (PMC12307169; doi:10.3389/fimmu.2025.1547888)
Supplement: Supplementary file 1 [file DataSheet1.docx]

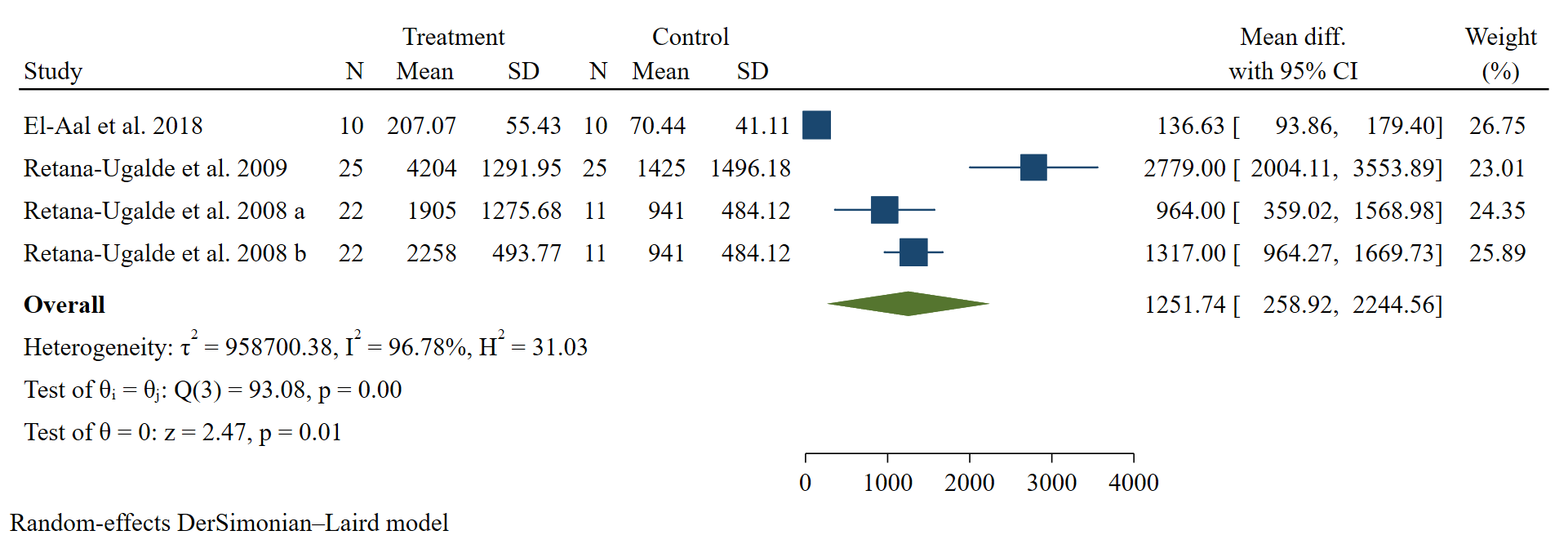


**Supplemental Figure 1**: The forest plot of the meta-analysis on the effect of vitamins E and C on plasma concentration of plasma GPx activity. Horizontal lines represent 95% CIs. Diamonds represent pooled estimates from random-effects analysis. CI: confidence interval


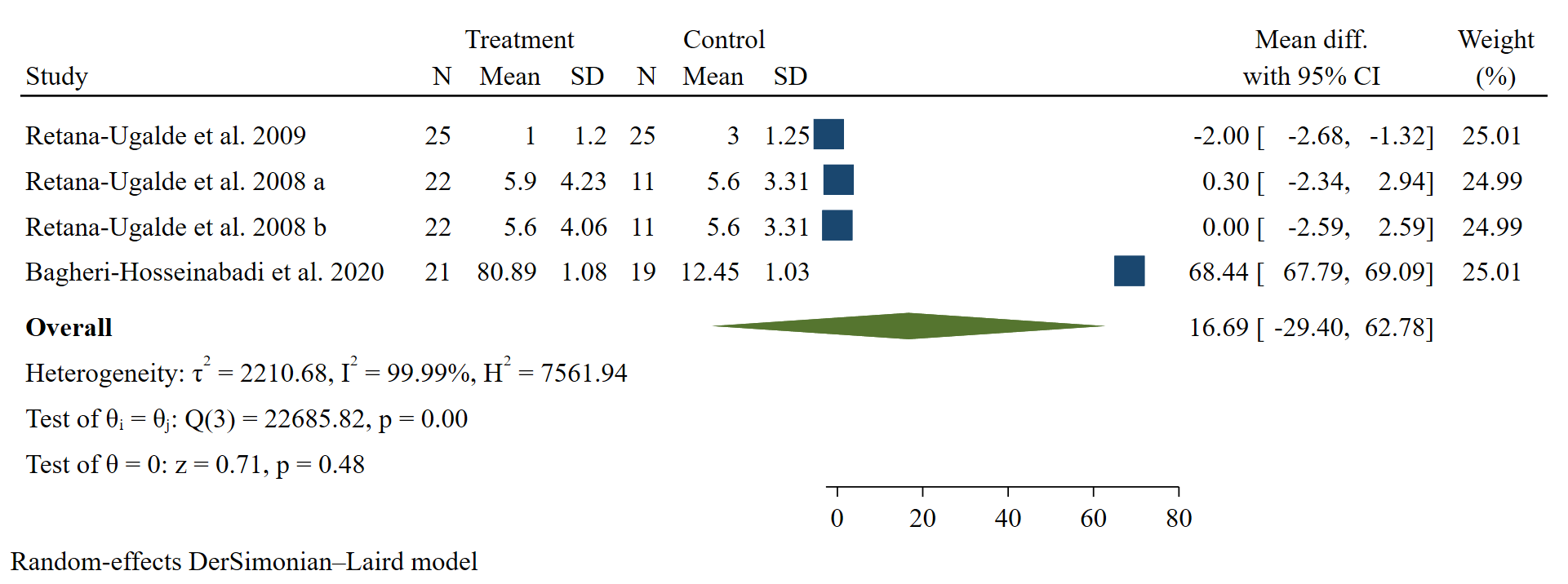


**Supplemental Figure 2**: The forest plot of the meta-analysis on the effect of vitamins E and C on plasma concentration of plasma SOD activity. Horizontal lines represent 95% CIs. Diamonds represent pooled estimates from random-effects analysis. CI: confidence interval
